# Literary Notes

**Published:** 1905-04

**Authors:** 


### LITERARY NOTES.

The *American Journal of Surgery and Gynecology,* formerly published at Saint Louis, has been sold to Dr. J. Macdonald, Jr., well known to the medical world as a successful journalist, who will publish it in New York under the name of the *American Journal of Surgery.* It will be edited by Dr. Walter M. Brickner, and the publication office is located at 92 William street.

J. B. Lippincott Company, Philadelphia, announce that they will publish during the present year a translation by Dr. Albion Walter Hewlett of the third German edition of the Principles of clinical pathology, by Dr. Ludolf Krehl, with an introduction by Dr. William Osler, of John Hopkins University. The work is well known in this country and in Europe as an authority upon the subjects treated, and has been copyrighted in the United States under the interim copyright act.

